# Cell-to-Cell Culture Inhibits Dedifferentiation of Chondrocytes and Induces Differentiation of Human Umbilical Cord-Derived Mesenchymal Stem Cells

**DOI:** 10.1155/2019/5871698

**Published:** 2019-11-16

**Authors:** Xingfu Li, Yujie Liang, Xiao Xu, Jianyi Xiong, Kan Ouyang, Li Duan, Daping Wang

**Affiliations:** ^1^Guangzhou Medical University, Guangzhou, Guangdong Province, China; ^2^Guangdong Provincial Research Center for Artificial Intelligence and Digital Orthopedic Technology, Shenzhen Key Laboratory of Tissue Engineering, Shenzhen Laboratory of Digital Orthopedic Engineering, Department of Orthopedics, Shenzhen Second People's Hospital (The First Affiliated Hospital of Shenzhen University, Health Science Center), Shenzhen 518035, Guangdong Province, China; ^3^Department of Chemistry, The Chinese University of Hong Kong, Shatin, Hong Kong SAR, China

## Abstract

**Background:**

Human umbilical cord-derived mesenchymal stem cells (hUC-MSCs) possess great promise as a therapeutic to repair damaged cartilage. Direct intra-articular injection of mesenchymal stem cells has been shown to reduce cartilage damage and is advantageous as surgical implantation and associated side effects can be avoided using this approach. However, the efficacy of stem cell-based therapy for cartilage repair depends highly on the direct interactions of these stem cells with chondrocytes in the joint. In this study, we have carried out an *in vitro* cell-to-cell contact coculture study with human articular chondrocytes (hACs) and hUC-MSCs, with the goal of this study being to evaluate interactions between hACs and hUC-MSCs.

**Methods:**

Low-density monolayer cultures of hUC-MSCs and hACs were mixed at a ratio of 1 : 1 in direct cell-to-cell contact groups. Results were analyzed using quantitative reverse transcription polymerase chain reaction (qRT-PCR), western blot, enzyme-linked immunosorbent assay (ELISA), and immunofluorescence.

**Results:**

A mixed coculture of hUC-MSCs and hACs was found to exhibit synergistic interactions with enhanced differentiation of hUC-MSCs and reduced dedifferentiation of chondrocytes. Mixed cultures after 21 days were found to exhibit sufficient chondrogenic induction.

**Conclusions:**

The results from this study suggest the presence of mutual effects between hUC-MSCs and hACs even culture at low density and provide further support for the use of intra-articular injection strategies for cartilage defect treatment.

## 1. Introduction

Repair of cartilage defects poses a large orthopedic challenge mainly due to the factor that the tissue has a limited intrinsic self-repair capacity. The trauma of articular cartilage is associated with articular surface defects, acute inflammation, and oxidative stress, while aging is accompanied by matrix degradation, chondrocyte apoptosis, and chronic inflammation. Following mature cartilage injury or aging, diseases such as osteoarthritis can arise. Development of novel tissue engineering strategies is of great importance in order to address cartilage repair. However, the effective treatment of cartilage defects represents a challenging problem within the field. Throughout the past two decades, there have been numerous advances towards the treatment of cartilage lesions [1]. Autologous chondrocyte implantation (ACI) is found to be the only FDA-approved cell-based therapy used for the treatment of cartilage defects, while the FDA-approved MACI (autologous cultured chondrocytes on porcine collagen membrane) is used as a more advanced treatment for the repair of symptomatic, full-thickness cartilage defects of the knee in adult patients on December 13, 2016. MACI is a next-generation approach to traditional ACI that provides the benefit of autologous cells and guided tissue regeneration using a biocompatible collagen scaffold. The MACI implant also has inherent advantages including surgical implantation via arthroscopy or mini-arthrotomy, the elimination of periosteal harvest, and the use of tissue adhesive in lieu of sutures [2]. Nowadays, there are 3 generations of ACI, but each one has its own shortages. The first generation of ACI is prone to periosteal hyperplasia due to the use of autologous periosteal covering, which requires secondary surgery and increases the risk of degeneration of new cartilage tissue. The second generation of ACI has the risk of cell leakage, uneven distribution, and collagen membrane shedding. The third generation of ACI requires high treatment costs, scaffold material, and long recovery time. However, the application of autologous chondrocytes has several disadvantages, limiting its potential as a clinical treatment [3]. These disadvantages include donor site morbidity and dedifferentiation of harvested chondrocytes following *ex vivo* monolayer expansion. The loss of phenotypic function during chondrocyte expansion in monolayer culture has become a serious challenge for the clinical expansion of autologous chondrocyte implantation (ACI) application [4]. Our past research studies and other research studies have demonstrated that following monolayer propagation of chondrocytes *in vitro*, which is often accompanied by chondrocyte dedifferentiation, collagen type I alpha 1 (Col1a1) expression was increased and collagen type II alpha 1 (Col2a1) expression was decreased [5, 6]. The exhibition of a fibroblast-like phenotype severely compromises the outcome of ACI and becomes a major obstacle for the widespread application of chondrocytes in cartilage defect repair [7, 8].

Recent studies have shifted from focusing on ACI to mesenchymal stem cell implantation (MSCI) therapy for articular cartilage repair [9–12]. Numerous regenerative medicine clinical trials or animal models have demonstrated that MSCI is a promising therapy for cartilage regeneration [13]. The advantages of MSCI therapy depend on the strong proliferation ability, low immunogenicity, and multidirectional differentiation potential of mesenchymal stem cells (MSCs). Specifically, evidence suggests that hUC-MSCs could serve as a promising source of cells for *in vivo* repair of cartilage defect [14]. This is due to their advantageous properties including noninvasive collection, high proliferative potential, lower immunogenicity, and chondrogenic potential *in vitro* [15–17]. Several animal studies and clinical studies have demonstrated that intra-articular injection of MSCs was safe and effective, which is effective for reducing pain, cartilage defects, and inflammation and improving knee function by regeneration of hyaline-like articular cartilage that results in long-term clinical and functional improvement of knee OA [18–23].

Following injection, MSCs distribute throughout the joint space and directly interact with any available surfaces of receptive cells and the microenvironment. Because cell fate is largely dependent on interactions between cells and multifactorial environmental cues, it is imperative that interactions between MSCs and hACs be understood in order to better predict therapeutic outcomes. Towards this end, numerous studies have investigated the effects of chondrocytes on MSCs *in vitro*, particularly in the context of MSC chondrogenesis. These studies demonstrated that, in coculture systems, chondrocytes enhance the chondrogenesis of MSCs [24, 25]. Likewise, our previous studies also demonstrated that indirect coculture in conditioned chondrocyte culture medium increases the expression of chondrogenic markers and induces differentiation of human umbilical cord blood-derived mesenchymal stem cells (hUCB-MSCs) into mature chondrocytes [26]. Bian and other researchers found that coculture also inhibits hypertrophic process during differentiation of MSCs [27, 28]. However, the effects of MSCs on hACs and on their ability to repair the extracellular matrix have been carried out few times [29, 30]. *In vitro* coculture of MSCs and chondrocytes represents a powerful approach to distinguish the contribution of each cell type and their interactions. Thus, in order to advance the field of cartilage regeneration, we must first understand the natural progression of repair prior to the identification of potential therapeutic targets. The aim of this research is to carry out an *in vitro* coculture of hACs and hUC-MSCs to shed light on the process of coculture. Specifically, we aim at understanding whether hACs can enhance hUC-MSCs chondrogenic differentiation, while also trying to understand the effects of cell-to-cell interactions on dedifferentiation in chondrocytes, in particular at the low-density culture. In addition, we aim at determining low-density seed cells and induction time of the two cell types. The successful application of a coculture technique to support cartilage formation will help to further demonstrate the value of intra-articular injections of hUC-MSCs in cartilage repair.

## 2. Methods

### 2.1. Preparation of hUC-MSCs

The study was carried out in full accordance with local ethical guidelines. Samples were collected after obtaining approval from the Ethics Committee of Shenzhen Second People's Hospital and written informed consent from healthy donors (25–28 years old, female) included in the study. According to the institutional guidelines, human umbilical cord (hUC) units were obtained from normal full-term and preterm deliveries without complications throughout pregnancy, in a physiological saline containing heparin anticoagulant, and were processed within 6 hr of collection. The units were stored and transported at 4°C. No complications were encountered upon hUC collection, and none of the samples had signs of coagulation or haemolysis. In brief, human umbilical cords were collected and cut into 3 to 5 cm pieces, and then vessels were removed from cord segments. Wharton's jelly was collected from hUC and cut into 2 to 3 mm^3^ tissue block and evenly placed in Petri dish with MesenGro® Human Mesenchymal Stem Cell Medium (StemRD, USA) that was supplemented with 10% fetal bovine serum (FBS; Gibco, Australia) and 10 *μ*g/L basic fibroblast growth factor (bFGF; Gibco, Australia). The tissue block was cultured at 37°C in a 5% CO_2_ incubator. Three days later, fresh medium was added to the flasks. Medium replacement was carried out every 72 h until the cells reached an 80% confluent layer. Cells were digested with 0.25% (w/v) trypsin plus 0.02% (w/v) EDTA (Hyclone, USA) and subcultured at a density of 1.0 × 10^4^ cells/cm^2^. Medium was changed twice a week. The hUC-MSCs of passage 3 (P3) were used for chondrocyte induction [25, 26].

### 2.2. Culture and Isolation of hACs

The collection of cartilage was approved by the Ethics Committee of Shenzhen Second People's Hospital. Samples were collected after obtaining written informed consent from all individuals. All the cartilage samples were obtained from volunteer donors (26–35 years old, male) after trauma patients, in a physiological saline system containing penicillin/streptomycin (P/S), and were processed within 6 hr of collection. For isolation of chondrocytes, the cartilage specimens were minced to 1 mm^3^ and digested in 1 mg/mL type II collagenase in Dulbecco's modified Eagle's medium (DMEM; Worthington Biochemical Corporation, USA) for 8 hr at 37°C in a shaker. After filtration, cells were harvested and seeded onto tissue culture flasks at a density of 1 × 10^4^ cells/cm^2^ and subcultured in chondrocyte growth medium (DMEM-F12, 10% FBS, 10 *μ*g/L bFGF, and 0.1 mg/mL P/S). All incubations occurred in a 5% CO_2_ atmosphere at 37°C. Medium was replaced 3 times a week until cells reached confluence. At 80% confluence, cells were harvested with a trypsin/EDTA solution (Gibco/Life Technologies, Australia) and seeded onto new flasks. Chondrocytes of passage 2 (P2) were used for chondrocyte induction [27].

### 2.3. Multiparametric Flow Cytometry Analysis of hUC-MSCs

Expression of cell surface markers on the hUC-MSCs was analyzed using flow cytometry. The following monoclonal antibodies (mouse anti-human) were used for flow cytometric immunophenotyping of human mesenchymal stem cells-extracellular vesicles (hMSCs-EV) and human mesenchymal stem cells (hMSCs): CD73 FITC, CD105 PE, CD45FITC, and CD34 PE (BD Bioscience, USA). The cells were characterized with regard to some positive MSC surface markers, including CD105 and CD73, and negative for CD34 and CD45 (hematopoietic markers). hUC-MSCs were suspended in PBS containing 5% bovine serum albumin (Sigma-Aldrich, USA) at a concentration of 3 × 10^5^ cells/50 *μ*L and stained with these markers, respectively. Labeled cells were acquired using a FACS flow cytometer (BD Biosciences, USA) for acquisition and analyzed using FlowJo Software (Tree Star) [26].

### 2.4. Differentiation Potential of hUC-MSCs

To characterize the differentiation capacity of hUC-MSCs into the various lineages like chondrogenic, adipogenic, and osteogenic cells, isolated hUC-MSCs were cultured in specific differentiation medium and simultaneously cultured in differentiation medium without growth factors serving as controls. When induction was completed, lineage-specific markers were analyzed by stain. Alizarin red stain of sulfated cartilage glycosaminoglycans (GAGs) demonstrated osteogenic induction. Accumulation of lipid-rich vacuole formation of the hUC-MSCs reveals that adipogenic induction was detected by intracellular Oil Red O stain. After 14 days of chondrogenic induction, cells were stained with toluidine blue. The positive acidic proteoglycan indicated the chondrocyte-like cell formation. After 14 days of induction, relative gene expressions were measured by quantitative reverse transcription polymerase chain reaction (qRT-PCR).

### 2.5. Coculture of hUC-MSCs and hACs

hACs (P2) and hUC-MSCs (P3) were used. For coculture with direct cell-to-cell contact, hACs and hUC-MSCs were mixed directly at a ratio of 1 : 1, and the area cell density for hUC-MSCs and hACs was 0.3 × 10^4^ (cells/cm^2^). All the cells were cultured in basal medium (DMEM-F12, 10% FBS, 100 U/ml penicillin, and 0.1 mg/ml streptomycin). The seeding cell number of hACs or hUC-MSC alone group used was 0.6 × 10^4^ (cells/cm^2^). For growth factor induction in monolayer culture, hUC-MSCs were maintained in basal medium supplemented with 0.1 mM dexamethasone, 40 mg/mL L-proline, 10 *μ*g/L transforming growth factor-beta-1 (TGF-*β*1; Peprotech, USA), 10 *μ*g/L insulin-like growth factor-1 (IGF-1; Peprotech, USA), 1% insulin transferrin selenium (ITS; Invitrogen, USA) [31–35]. Controls were monocultures of hACs and hUC-MSCs alone with basal medium. All cells were incubated for 28 days at 37°C in a humidified atmosphere of 5% CO_2_ and the medium was changed every three days.

### 2.6. Immunofluorescence Stain of Type II Collagen

Hyaline cartilage marker protein type II collagen (COL2) was examined using immunofluorescence stain [36]. All groups were harvested 28 days after seeding. The samples were fixed in 4.0% formaldehyde in PBS for 15 min at room temperature, followed by washing with PBS and permeabilization with 0.1% Triton X-100 for 10 min (Sigma-Aldrich, USA). Then cells were blocked in 5% bovine serum albumin at RT. Then cells were subsequently incubated overnight at 4°C with mouse polyclonal antibodies (R&D System, USA) against COL2 at 1 : 100 dilutions overnight, rinsed with PBS three times for 5 min each, and then incubated with an Alexa Fluor®594-conjugated goat anti-mouse IgG secondary antibody (Molecular Probes, USA) at 1 : 200 dilutions for 1 hr. After rinsing three times with PBS, nuclei were counterstained with 4,6-diamidino-2-phenylindole DAPI (Molecular Probes, USA) for 10 min and then rinsed with PBS. Subsequently, the fluorescent signal of cell nuclei and COL2 was visualized under wavelengths of 594 nm and 405 nm, respectively, using an inverted microscope (Olympus, Japan) equipped with a digital camera (Olympus, Japan) [37].

### 2.7. RNA Extraction and qRT-PCR

Samples in each group were harvested on days 14, 21, and 28 (*n* ≥ 3 per group). RNA was extracted from cell samples using TRIzol reagent (Invitrogen, USA) according to the manufacturer's instructions. cDNA was synthesized from total RNA using an Omniscript RT kit (Qiagen, Germany). The mRNA expression levels of SRY-Box 9 (SOX9), Col1a1, and Col2a1 were determined by qRT-PCR using SYBR Premix EX Taq (Takara, Japan). The forward and reverse primer pairs are shown in Table 1. To normalize mRNA levels, the GAPDH housekeeping gene was used as an internal control [38]. The level of expression was then calculated as 2^−ΔΔCt^ and expressed as the mean. The results are presented as mean fold change relative to control sample.

### 2.8. Western Blot

The expression levels of SOX9, COL2, and type I collagen (COL1) proteins from cell samples were analyzed as described previously [39]. Cells were lysed in lysis buffer (50 mM Tris-HCl, pH 7.4, 150 mM NaCl, 1% NP-40, and 0.1% sodium dodecyl sulfate) supplemented with protease inhibitor cocktail set I (Biotool, Jupiter, FL, USA) and phenylmethanesulfonyl fluoride (PMSF; Sigma-Aldrich, USA). Samples with equal protein concentration (about 80 *μ*g/lane) were subjected to SDS-PAGE and transferred to a PVDF membrane. Blots were blocked with 5% skim milk/TBS-Tween 20 for 1 hr at room temperature and probed with primary antibodies: mouse anti-SOX9 (Santa Cruz Biotechnology, USA), mouse anti-COL1 (Abcam, UK), mouse anti-COL2 (Abcam, UK), and mouse anti-*β*-actin (Abcam, UK) overnight at 4°C. Then, blots were washed with PBS-Tween 20 (0.1%) and incubated with horseradish peroxidase-conjugated secondary antibodies (1 : 1000) for 1 hr at room temperature.

### 2.9. ELISA

The TGF-*β*1 concentration in the supernatant was determined by a human TGF-*β*1 ELISA kit (R&D system, USA). BCA quantifying the total protein was conducted before ELISA and then the same protein quantity was loaded from different culture conditions. Absorbance was measured at a wavelength of 450 and 550 nm. The 450 nm values were subtracted by the 550 nm values for the correction of optical imperfections [40].

### 2.10. Statistical Analysis

Data were expressed as the mean ± standard deviation. Differences between groups were examined for statistical significance using one-way analysis of variance using SPSS statistical analytical software (ver. 18.0; IBM, USA). *p* values less than 0.05 were denoted the presence of a significant difference between groups.

## 3. Results

### 3.1. Identification of hUC-MSCs

Following isolation of mesenchymal cells according to the adherence criteria, the third passage of hUC-MSCs was analyzed in order to confirm their identity. MSCs are known not to express CD34, CD45, CD117 (cKit), HLA class I, and HLA-DR antigens, whereas they are known to express CD13, CD29, CD44, CD73, CD90, CD105, and CD166. Surface markers considered to be positive for the mesenchymal cell lineage (CD73 and CD105) and negative for the hematopoietic lineage (CD34 and CD45) were used for the characterization of hUC-MSCs. Flow cytometry analysis revealed that the isolated hUC-MSC population exhibited high expression levels of both CD105 and CD73 and low expression levels of CD34 and CD45. In addition, these cells were found to have acquired fibroblastic morphology that is characteristic of MSCs. These results therefore confirmed the existence of MSCs in hUC. The proportion of stem cells was found to meet the identification criteria [26].

### 3.2. Multilineage Differentiation Potential of hUC-MSCs

To characterize differentiation potential of hUC-MSCs, the isolated hUC-MSCs were cultivated in osteogenic, adipogenic, and chondrogenic inducing media. For chondrogenic differentiation potential, hUC-MSCs were cultured for 14 days in chondrogenic medium, compared to their respective undifferentiated cultures (Supplementary [Supplementary-material supplementary-material-1]). After 14 days in osteogenic medium, alizarin red stain of sulfated cartilage GAGs demonstrated that mineralized nodules were formed in the hUC-MSCs after 14 days under the osteogenic induction (Supplementary [Supplementary-material supplementary-material-1]). After 14 days of cultivation in adipogenic medium, the intracellular lipid-rich vacuole formation in hUC-MSCs was observed by Oil Red O stain (Supplementary [Supplementary-material supplementary-material-1]). These data proved multipotent differentiation of hUC-MSCs into trilineages, which could provide stem cell for coculture studies.

To determine the respective differentiation capacities, hUC-MSCs were exposed to osteogenic, adipogenic, or chondrogenic differentiation conditions and relative gene expressions were detected by qRT-PCR. As compared to that in hUC-MSCs cultured in specific differentiation medium, expression of chondrogenic specific genes aggrecan (ACN), Col2a1, and SOX9, osteogenic genes osteocalcin and Runt-related transcription factor-2 (Runx2), and adipogenic gene Primary Productivity Algorithm Round Robin (PPARr) were significantly lower in the control group (Supplementary [Supplementary-material supplementary-material-1]).

### 3.3. Coculture with hUC-MSCs Inhibited Chondrocyte Dedifferentiation

The *in vitro* monolayer propagation of chondrocytes could cause the cells to dedifferentiate back to exhibit a fibroblast-like phenotype, especially with higher passage numbers. Therefore, it is important to gain a better understanding regarding environmental effects on the phenotype of the chondrocytes prior to using intra-articular injection of MSCs for further applications.

**Figure 1:**
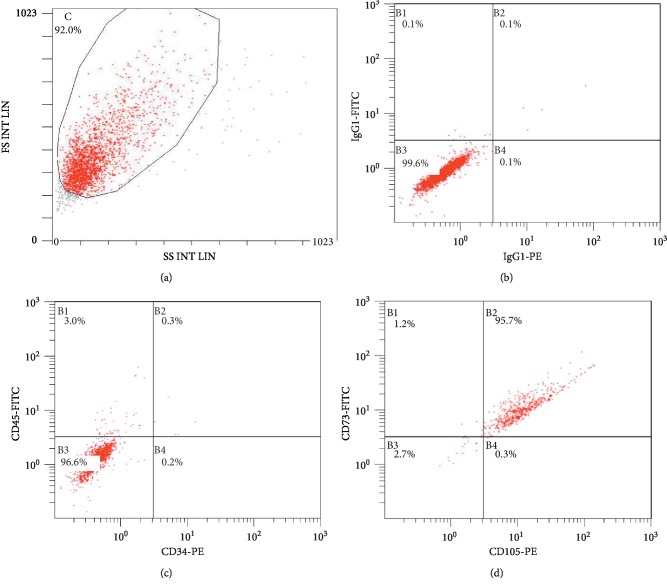
Characterization of hUC-MSCs by flow cytometry. (a) Representative dot plots showing the FSC to SSC. (b) IgG1-PE and IgG1-FITC were used as controls. (c) Cells expressing both CD34 and CD45 represented 0.7% of the population. (d) Cells expressing both CD105 and CD73 accounted for 96.4% of the population.

In order to detect the biological changes surrounding the dedifferentiation phenotypic changes, we used the dedifferentiation marker, chondrocyte-related gene Col1a1. The qRT-PCR results demonstrated that the mRNA expression of Col1a1 was increased with prolonged passaging of chondrocytes, while the gene expression level of Col2a1 was decreased during chondrocyte monolayer culture (Figure 1(a)). These results suggest that many of the cell dedifferentiation is due to the presence of monolayer culture.

**Figure 2:**
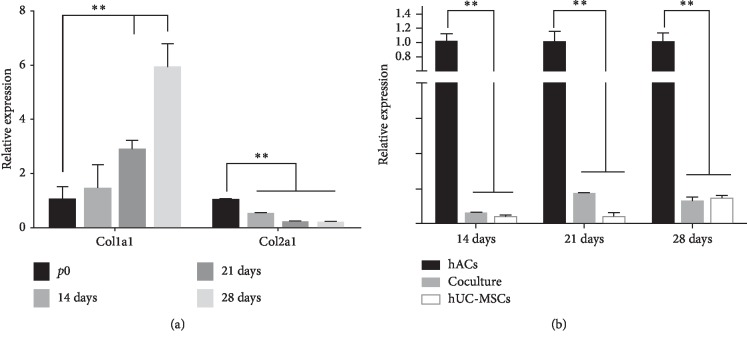
(a) qRT-PCR showed that, with prolonged passaging, the mRNA expression level of Col1a1 was increased, while the expression level of Col2a1 was decreased during chondrocyte monolayer culture. (b) Effects of direct coculture of hUC-MSCs with hACs (50 : 50) on gene expression of Col1a1 after 14 days, 21 days, and 28 days.

By using hACs as control, our results demonstrated that Col1a1 expression was greatly inhibited in the direct coculture group (Figure 1(b)). Transcript results from the qRT-PCR analysis provided the desired results. While the hAC monolayer was found to demonstrate the most dedifferentiation, cell-to-cell interactions showed the least dedifferentiation *in vitro*. A significant downregulation of Col1a1 gene expression was observed when hUC-MSCs were cocultured with hACs (50 : 50). Meanwhile, upregulation of SOX9 expression could also contribute to the maintenance of the chondrocyte phenotype and the inhibition of chondrocyte dedifferentiation (Figure 2(b)).

### 3.4. Coculture with hACs Induced Chondrogenesis of hUC-MSCs

**Figure 3:**
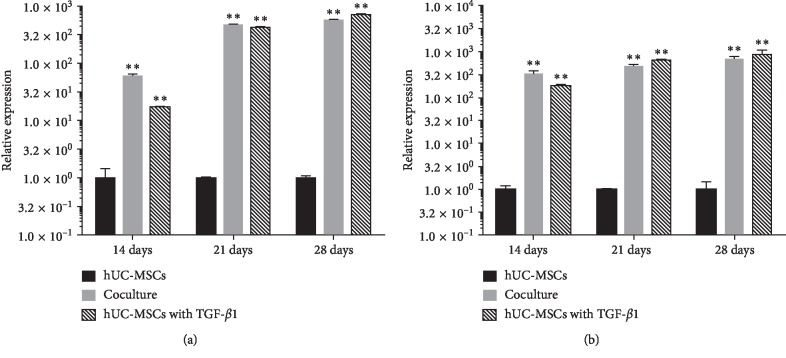
Quantitative gene expression levels for Col2a1 (a) and ACN (b). hUC-MSC mixed culture with hACs at a ratio of 1 : 1 for 14 days, 21 days, and 28 days, respectively. ^*∗*^*p* < 0.05; ^*∗∗*^*p* < 0.001.

Serviced as specific and noncharacteristic for cartilage engineering applications, TGF-*β*1 (10 ng/ml) is known to induce chondrogenic differentiation for the upregulation of the expression of several markers, including SOX9, ACN, and Col2a1. A significant upregulation of Col2a1 gene expression between days 14 and 28 was detected in TGF-*β*1-treated hUC-MSC monocultures. In order to determine whether hACs affect the gene expression levels of extracellular matrix macromolecules (ECMs) in hUC-MSCs, we analyzed the mRNA expression levels of the chondrogenic differentiation marker, Col2a1, at days 14, 21, and 28 using qRT-PCR. At day 14, we observed a significant increase in Col2a1 gene expression in all cocultured regimens in comparison with monocultures of only hACs or hUC-MSCs. This increase of Col2a1 gene expression was detected in all cocultured regimens from day 14 to day 28 (Figure 3). However, we found that, in some groups, Col2a1 gene expression was downregulated on day 28 compared to day 21 in mixed hAC and hUC-MSC cocultures. We found that the mixture group exhibited much better performance. In the case of hUC-MSC coculture, we detected a highly significant upregulation of Col2a1 gene expression from days 14 to 28, suggesting that hACs support chondrogenic differentiation.

### 3.5. Coculture of hACs Induced Transcription Factor SOX9

**Figure 4:**
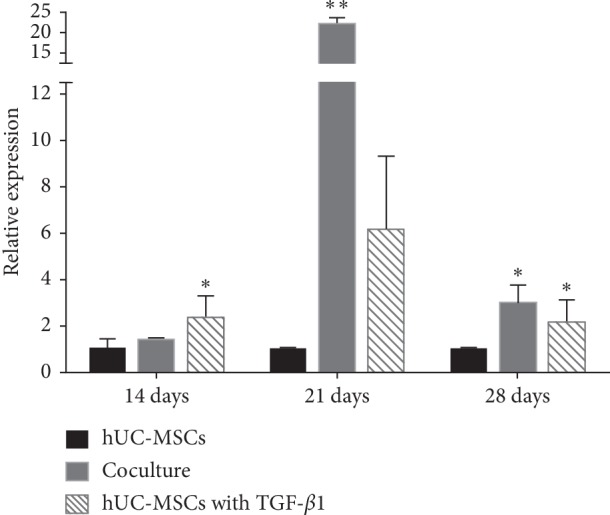
Quantitative gene expression levels for SOX9 mixed culture with chondrocytes at a ratio of 1 : 1 for 14 days, 21 days, and 28 days, respectively. Undifferentiated hUC-MSCs were also cultured as monolayers and used as a control. ^*∗*^*p* < 0.05; ^*∗∗*^*p* < 0.001.

During *in vitro* chondrogenesis of hUC-MSCs, the transcription factor SOX9 is known to promote transcription of genes encoding cartilage matrix proteins, including COL2 and ACN. Treatment with growth factors was found to upregulate SOX9 expression levels. Therefore, we studied the expression levels of the transcription factor, SOX9, in association with a commitment of MSCs to the chondrocytes. We demonstrated a chondrocyte phenotype with an increased expression of SOX9 in the presence of growth factors. We also showed that our coculture model allows for MSC commitment to chondrogenesis, showing high expression of SOX9 during chondrogenic differentiation (Figure 4). Thus, we show that the upregulation of the transcription factor SOX9 will promote coculturing of hUC-MSCs with mature chondrocytes to induce differentiation.

### 3.6. Immunofluorescence Stain of Collagen

**Figure 5:**
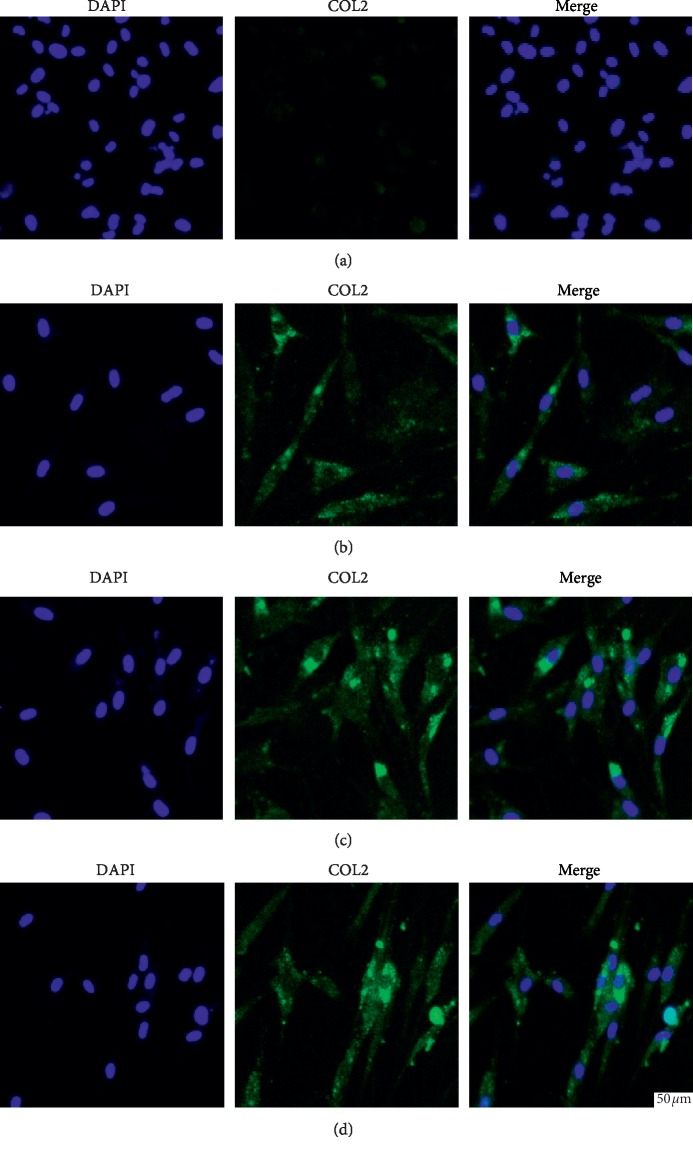
COL2 protein levels were characterized by immunofluorescence. Immunofluorescence stain of COL2 is shown in green. (a) hUC-MSCs alone; (b) direct coculture (50 : 50) at 14 days; (c) direct coculture (50 : 50) at 21 days; (d) direct coculture (50 : 50) at 28 days. Nuclei were stained with DAPI (4′,6-diamidino-2-phenylindole, blue color). Scale bar is 50 *μ*m.

Immunofluorescence stain was carried out in order to directly visualize collagen synthesis following 28 days of coculture of hUC-MSCs with hACs to induce chondrogenic differentiation in culture. Images showed that in the presence of TGF-*β*1, COL2 protein stain was more marked with a preferential localization to the ECM. Stain experiments revealed that COL2 protein was minimally detectable via immunofluorescence stain in cultures containing only hUC-MSCs, while both growth factor differentiated hUC-MSCs and direct cocultures of hUC-MSCs with hACs exhibited detection of COL2 protein stain. In addition, the direct coculture group exhibited a more marked fluorescence than the TGF-*β*1-induced group (Figure 5). The direct coculture groups showed similar stain in different coculture periods. The hUC-MSCs cultured alone exhibited the weakest fluorescent signal amongst all of the experimental groups. This indicated that these progenitor cells do not undergo significant chondrogenesis in the absence of external signals. In conclusion, immunofluorescence stain analyses confirmed that chondrogenic differentiation is primarily induced in the presence of direct coculture, whereas only modest COL2 expression is observed in the absence of growth factors.

### 3.7. Analysis of Chondrocyte and Cartilage Matrix-Related Protein Production

**Figure 6:**
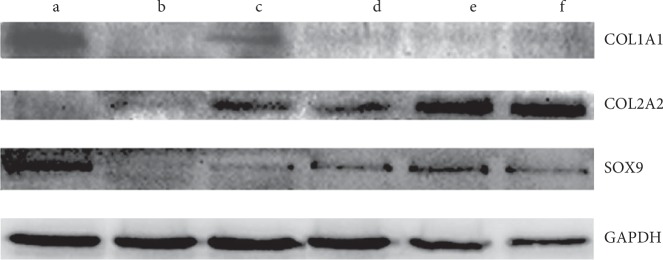
SOX9, COL2, and COL1 protein levels were detected by western blot. (a) hACs alone; (b) hUC-MSCs alone; (c) hUC-MSCs cultured with growth factors; (d) direct coculture (50 : 50) at 14 days; (e) direct coculture (50 : 50) at 21 days; (f) direct coculture (50 : 50) at 28 days.

In order to further evaluate chondrogenesis in all groups, the chondrogenesis marker proteins, COL2 and SOX9, were analyzed by western blot. Following 28 days of *in vitro* culture, SOX9 and COL2 proteins were found to be upregulated in direct cocultures relative to hUC-MSC monocultures with growth factors (Figure 6). This suggests that coculture promotes the mature form of the collagen. In addition, protein levels of the dedifferentiation maker, COL1, detected by western blot exhibited low levels in coculture groups, while COL1 protein was more abundant in hAC monoculture compared to direct cell-cell contact cocultures.

### 3.8. ELISA Quantification of TGF-*β*1

Secreted TGF-*β*1 expression levels in the medium were measured using ELISA following 28 days of *in vitro* culture. Secreted TGF-*β*1 was found to be significantly upregulated in the direct coculture groups relative to hUC-MSC monoculture. However, TGF-*β*1 levels in the direct coculture groups were found to be lower than in the hUC-MSC monoculture supplemented with growth factors (*p* < 0.05). TGF-*β*1 level in hAC monoculture was found to be the highest out of all the groups (*p* < 0.05), while the hUC-MSC monoculture had the lowest TGF-*β*1 level (*p* < 0.05). Interestingly, the increase of hUC-MSCs in the coculture groups was found to correlate with a decrease in TGF-*β*1 concentration (Supplementary [Supplementary-material supplementary-material-1]).

## 4. Discussion

For the *in vitro* generation of neocartilage, the use of hUC-MSCs possesses numerous advantages, including a convenient collection method, an increased retention of their multipotency over the course of several passages, and reduced immunogenicity. Thus, hUC-MSCs are a promising source of cells as a stem cell therapy treatment of cartilage defects. One challenge that exists in applying hUC-MSCs for cartilage repair is the differentiation of hUC-MSCs into chondrocytes. Increased research is focused on understanding the controlled and induced differentiation of MSCs into chondrocytes. Conventional strategies to differentiate MSCs using exogenous inductive molecules, such as transforming growth factor (TGF-*β*1 and *β*3), fall short of satisfying the needs of clinical applications as it results in the generation of differentiated cells that exhibit a hypertrophic phenotype and subsequently extensive calcification of endochondral bone formation [25].

Numerous earlier studies have demonstrated that a variety of cytokine factors secreted by articular chondrocytes function to enhance the chondrogenesis of MSCs as well as inhibit hypertrophy of MSCs [32, 33]. This promotes the coculture between MSCs and chondrocytes and has been thought to help overcome this ongoing issue of chondrocyte differentiation. However, most research studies used the density at 2.4 × 10^6^ cells/cm^2^ [34, 35], and they barely exhibit the primary culture of MSCs at low densities (≤10^4^ cells/cm^2^) for 1 month. We knew that the lower the cell density is, the more difficult it is to maintain the cells in culture. In this study, we adopted the low density of hUC-MSCs at 0.6 × 10^4^ cells/cm^2^, about 400-fold lower than other coculture research studies. Our previous work suggests that chondrogenesis of human umbilical cord blood-derived mesenchymal stem cells (hUCB-MSCs) can occur in combination with hACs in direct and indirect coculture, even at low density [26, 35]. In the present study, we demonstrate that coculture could also stimulate the differentiation of hUC-MSCs into chondrocytes. The chondrogenic markers, COL2 and SOX9, of the coculture group (hUC-MSCs/hACs, 50 : 50) showed the highest level of expression in comparison with the MSC monoculture alone group. Our studies showed that low-density coculture could induce hUC-MSC differentiation into chondrocytes.

More importantly, our data highlight a strategic role of MSCs in inhibiting the dedifferentiation into fibrocartilage in cartilage remodeling. We found that the upregulation of Col1a1 or the downregulation of Col2a1 could result in the decreased ratio of COL2 to COL1, a classical index of chondrocyte dedifferentiation. In the coculture environment, Col1a1 expression levels were found to be decreased during chondrocyte passage, suggesting that hUC-MSCs help to reduce fibrochondrocyte and could play a role in improving cartilage function.

*In vitro* direct coculture was found to be able to be used to imitate the *in vivo* mutual effect. This study suggests that the *in vivo* injection of stem cell can direct the fate of stem cells towards tissue-specific progenitors with high therapeutic potential. Over the past few years, the effectiveness of stem cell injections has been demonstrated in the treatment of knee osteoarthritis. Intra-articular injection of hMSCs has been shown to enhance expression levels of rat type II collagen (rat-COL2), reduce inflammation, and inhibit osteoarthritis progression [37]. While stem cell therapeutics hold great promise, the ability to obtain optimal cell populations for transplantation and chondrogenic induction remains a major obstacle. However, in contrast to autologous transplanted chondrocytes that have the tendency to mature into cartilage, *in vivo* injected MSCs were found to preferentially differentiate into bone tissue [38, 39]. As the intra-articular injection of MSCs will inevitably result in the MSCs in direct contact with chondrocytes that reside in the surface of joint, direct MSC-chondrocyte cell contact is most likely responsible for the effect of cartilage repair. However, the stem cell destinations, final differentiation fate, and chondrocyte response is not easy to track accurately and noninvasively. Thus, this research provides the field with great value in that chondrocyte/MSC coculture model directs the path towards an effective emulation and decomposition *in vivo* injection of stem cell conditions. As for the development of stem cell-based therapy for cartilage regeneration for repairs, interactions between stem cells and the local chondrocytes should be taken into consideration to provide an optimal microenvironment for stem cell chondrogenesis, while maintaining the chondrocyte phenotype. We highlight that the number of stem cells that are injected into the cartilage is an important consideration in tissue engineering. It appears that a coculture group shows much better results than any other group. Additionally, our findings demonstrate that a 21-day culture period is necessary and sufficient to change production in direct coculture status.

While the bidirectional interactions between MSCs and articular chondrocytes have been recently noted, the mechanisms by which culture inhibits the dedifferentiation of chondrocytes and induces differentiation of hUC-MSCs are not well understood. Articular chondrocytes possess a desired cartilage phenotype and have the ability to secrete abundant cartilaginous extracellular matrix that may stimulate chondrogenesis of MSCs. In addition, MSCs also possessing a candidate secreted factor or anti-inflammatory potential could be responsible for improved chondrocyte phenotypes. In this study, we have investigated one potential mechanism by which transcript factor or growth factor shear regulates chondrocyte differentiation. Our results demonstrate that total TGF-*β*1 levels were significantly higher in coculture status along with days. We also show that upregulation of the transcript factor, SOX9, is partially mediated by TGF-*β*1 during chondrocyte differentiation [40]. Thus, crosstalk between growth factors and transcript factors is implicated in the bidirectional interactions between MSCs and hACs.

Thus, next-generation stem cell-based therapeutics for the treatment of cartilage defects must continue to explore the mechanisms of coculture systems that may function to enhance chondrogenesis and balance the effects of different coculture conditions to achieve an optimal tissue product. Overall, our current study provides a basis for improved protocols to direct hUC-MSC differentiation towards the development of cartilage tissue replacements suitable for implantation.

Although many found that a major concern of intra-articular injection of MSC suspensions is the extremely low anchorage/attachment rate, people gradually realized that potential of stem cell therapy depends largely on the ability of stem cell to adjust receptor cells by cell signaling. For example, this study confirmed that hUC-MSCs could inhibit dedifferentiation of chondrocytes and other scholars have confirmed that MSCs could inhibit inflammation in articular cavity, in addition to their ability to locate the site of injury and differentiate into target cells. Therefore, the signal transduction between MSCs and chondrocytes needs to be further studied. In addition, this study should carry out animal experiments to verify the inhibitory effect of MSCs on dedifferentiation of chondrocytes in vivo and explore the influence of the intra-articular environment on MSCs, so as to explore the interaction between MSCs and chondrocytes, and the mode of cellular signal transduction.

## 5. Conclusions

Our data support the idea that hUC-MSCs possess a greater capacity for chondrogenesis and are able to synthesize extracellular matrix while undergoing coculture with mature chondrocytes isolated from articular cartilage. When cocultured with hUC-MSCs, biosynthesis of COL2 and SOX9 and a reduction in Col1a1 expression will reverse the dedifferentiated procedure and support the chondrocyte phenotype.

In summary, our study has demonstrated that interactions between hUC-MSCs and hACs at close proximity show a mutual benefit through the enhancement of cartilage matrix production and the inhibition of dedifferentiation. However, more basic and preclinical studies that consider direct intra-articular injection of MSCs as an alternative treatment are needed. Such studies will provide the simplest cell-based strategies for cartilage regeneration.

## Figures and Tables

**Table 1:** Primer sequences used for qRT-PCR.

Genes	Forward primer (5′–3′)	Reverse primer (5′–3′)
*SOX9*	GACGTGCAAGCTGGGAAA	CGGCAGGTATTGGTCAAACTC
*Col2a1*	CGCCACGGTCCTACAATGTC	GTCACCTCTGGGTCCTTGTTCAC
*Col1a1*	GACATGTTCAGCTTTGTGGACCTC	GGGACCCTTAGGCCATTGTGTA
*GAPDH*	GGCACAGTCAAGGCTGAGAATG	ATGGTGGTGAAGACGCCAGTA

## Data Availability

In this study, all the data were obtained from the Shenzhen Key Laboratory of Tissue Engineering. The data used to support the findings of this study are included within the article.

## Supplementary Materials

Supplementary Materials Supplementary Figure 1: chondrogenic differentiation, osteogenic differentiation, and adipogenic differentiation of isolation of hUC-MSCs. Supplementary Figure 2: after 14 days of reliable induction, the osteogenic genes Runx2 and osteocalcin, adipogenic gene PPARr, and chondrogenic specific genes ACN, Col2a1, and SOX9 detected by RT-PCR. Supplementary Figure 3: TGF-*β*1 secreted into the medium measured using ELISA.
