# Supplementary material for: Cell-to-Cell Culture Inhibits Dedifferentiation of Chondrocytes and Induces Differentiation of Human Umbilical Cord-Derived Mesenchymal Stem Cells
Source: Biomed Res Int. 2019 Nov 16;2019:5871698. doi: 10.1155/2019/5871698 (PMC6885164; doi:10.1155/2019/5871698)
Supplement: Supplementary Materials — Supplementary Figure 1: chondrogenic differentiation, osteogenic differentiation, and adipogenic differentiation of isolation of hUC-MSCs. Supplementary Figure 2: after 14 days of reliable induction, the osteogenic genes Runx2 and osteocalcin, adipogenic gene PPARr, and chondrogenic specific genes ACN, Col2a1, and SOX9 detected by RT-PCR. Supplementary Figure 3: TGF-β1 secreted into the medium measured using ELISA. [file 5871698.f1.pdf]

**Additional files**

Supplementary Figure 1:

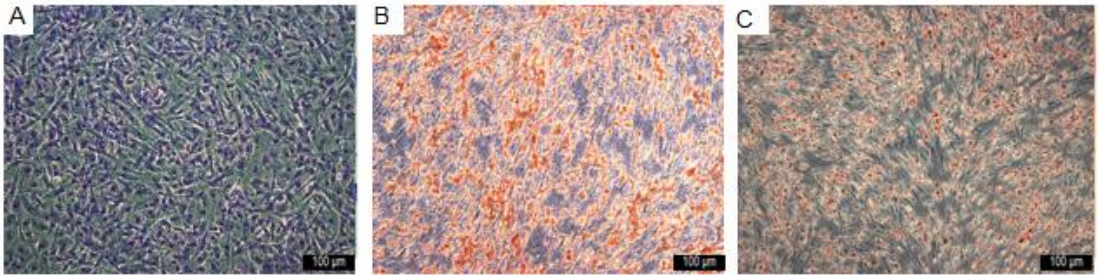


Figure S 1. Chondrogenic differentiation, osteogenic differentiation and adipogenic differentiation of isolated of hUC-MSCs. A, After 14 days of chondrogenic induction, hUC-MSCs were stained by toluidine blue; B, After 14 days of osteogenic induction, hUC-MSCs were stained by Alizarin red; C, After 14 days of adipogenic induction, hUC-MSCs were stained by Oil Red O. Scale bar=100μm.

Supplementary Figure 2:

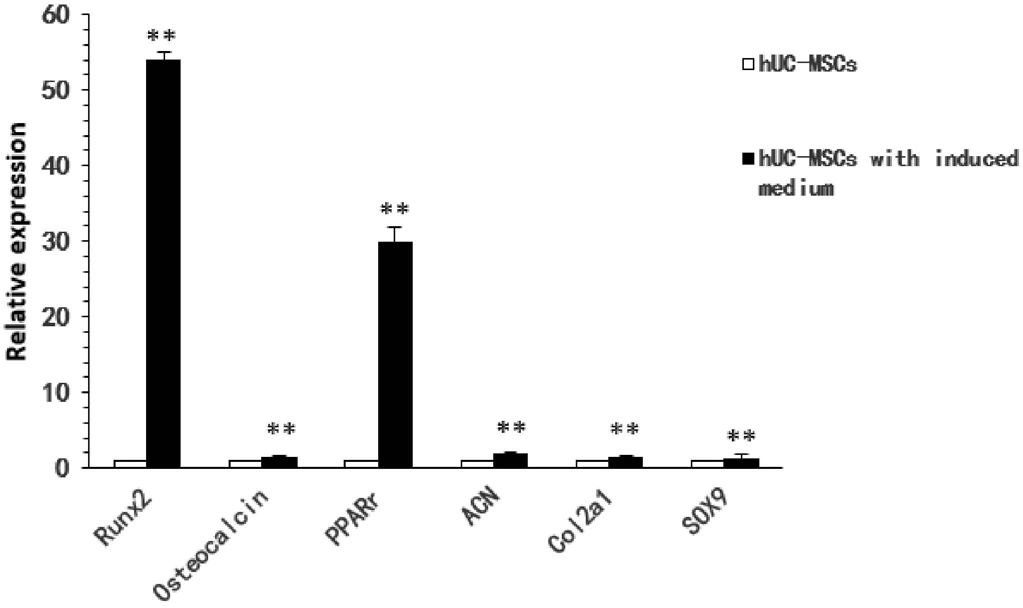


Figure S 2. After 14 days of respective induction, the osteogenic gene Runx2 and Osteocalcin, adipogenic gene PPARr and chondrogenic specific gene ACN, Col2a1 and SOX9 were detected by qRT-PCR. The relative gene expressions were normalized to GAPDH and calculated by $2^{-\Delta\Delta Ct}$ method.

Supplementary Figure 3:

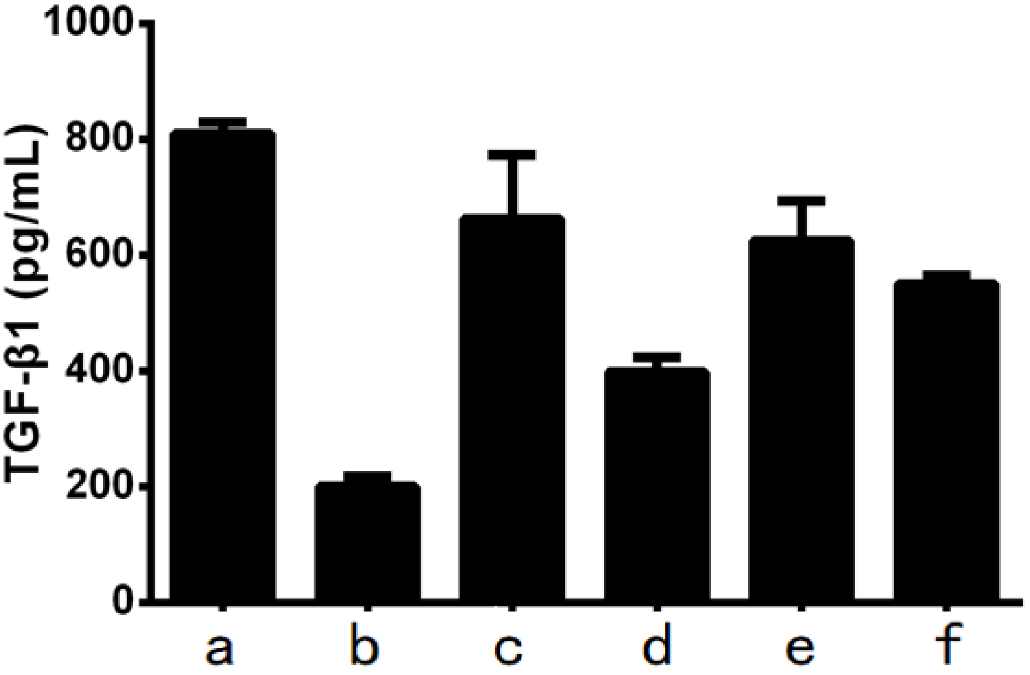


Figure S 3. TGF-β1 secreted into the medium was measured using ELISA. Statistically significant differences were found by one-way ANOVA in TGF-β1 concentration. a, hACs alone; b, hUC-MSCs alone; c, hUC-MSCs cultured with growth factors; d, Direct co-culture (50:50) after 14 days; e, Direct co-culture after 21 days; f, Direct co-culture after 28 days.
